# Performance of a trigger tool for detecting adverse drug reactions in patients with polypharmacy acutely admitted to the geriatric ward

**DOI:** 10.1007/s41999-022-00649-x

**Published:** 2022-05-30

**Authors:** Nikki M. F. Noorda, Bastiaan T. G. M. Sallevelt, Wivien L. Langendijk, Toine C. G. Egberts, Eugène P. van Puijenbroek, Ingeborg Wilting, Wilma Knol

**Affiliations:** 1 Geriatric Medicine Department, University Medical Centre Utrecht, Heidelberglaan 100, 3508 GA Utrecht, the Netherlands; 2 Clinical Pharmacy Department, University Medical Centre Utrecht, Utrecht, the Netherlands; 3 Division Pharmacoepidemiology and Clinical Pharmacology, Utrecht Institute for Pharmaceutical Sciences (UIPS), Faculty of Science, Utrecht University, Utrecht, the Netherlands; 4 The Netherlands Pharmacovigilance Centre Lareb, ‘s-Hertogenbosch, the Netherlands; 5 Division of PharmacoTherapy, -Epidemiology and -Economics, University of Groningen, Groningen, the Netherlands

**Keywords:** Geriatric medicine, Adverse drug events, Adverse drug reactions, Medication safety, Polypharmacy

## Abstract

**Purpose:**

Adverse drug reactions (ADRs) account for 10% of acute hospital admissions in older people, often under-recognised by physicians. The Dutch geriatric guideline recommends screening all acutely admitted older patients with polypharmacy with an ADR trigger tool comprising ten triggers and associated drugs frequently causing ADRs. This study investigated the performance of this tool and the recognition by usual care of ADRs detected with the tool.

**Methods:**

A cross-sectional study was performed in patients ≥ 70 years with polypharmacy acutely admitted to the geriatric ward of the University Medical Centre Utrecht. Electronic health records (EHRs) were screened for trigger–drug combinations listed in the ADR trigger tool. Two independent appraisers assessed causal probability with the WHO-UMC algorithm and screened EHRs for recognition of ADRs by attending physicians. Performance of the tool was defined as the positive predictive value (PPV) for ADRs with a possible, probable or certain causal relation.

**Results:**

In total, 941 trigger–drug combinations were present in 73% (*n* = 253/345) of the patients. The triggers fall, delirium, renal insufficiency and hyponatraemia covered 86% (*n* = 810/941) of all trigger–drug combinations. The overall PPV was 41.8% (*n* = 393/941), but the PPV for individual triggers was highly variable ranging from 0 to 100%. Usual care recognised the majority of ADRs (83.5%), increasing to 97.1% when restricted to possible and certain ADRs.

**Conclusion:**

The ADR trigger tool has predictive value; however, its implementation is unlikely to improve the detection of unrecognised ADRs in older patients acutely admitted to our geriatric ward. Future research is needed to investigate the tool’s clinical value when applied to older patients acutely admitted to non-geriatric wards.

**Supplementary Information:**

The online version contains supplementary material available at 10.1007/s41999-022-00649-x.

## Key summary points

**Aim:**

To investigate the performance of an adverse drug reaction (ADR) trigger tool in patients with polypharmacy acutely admitted to our geriatric ward.

**Findings:**

The ADR trigger tool had a positive predictive value (PPV) of 41.8%. Usual care recognised 83.5% of ADRs considered as possible, probable or certain, increasing to 97.1% when restricted to probable and certain ADRs.

**Message:**

It is unlikely that implementation of the ADR trigger tool will improve detection of unrecognised ADRs in older patients acutely admitted to our geriatric ward.

**Supplementary Information:**

The online version contains supplementary material available at 10.1007/s41999-022-00649-x.

## Background

Older people are more susceptible to adverse drug reactions (ADRs) due to comorbidity, polypharmacy, frailty and age-related changes in pharmacokinetics and -dynamics [1–3]. It is estimated that ADRs account for approximately 10% of all acute hospital admissions in older people [4, 5]. Despite this high frequency of hospital admissions due to ADRs in older people, studies show that drug-related problems, including ADRs, are missed or misdiagnosed by physicians at the emergency department in approximately 40–60% of the cases [6–8]. Consequently, methods to improve detection and management of ADRs are needed [9].

Polypharmacy is one of the most important risk factors for developing ADRs [10]. It is known that a few commonly used drug classes account for the majority of ADRs leading to or developed during hospital admission in the older population [1, 3–5, 9]. A meta-analysis found that ADR-induced hospital admissions were most frequently related to non-steroidal anti-inflammatory drugs (NSAIDs) causing upper gastrointestinal bleeding, hypertension, coronary events and renal failure. Other ADRs frequently associated with hospitalisations were hypotension due to beta-blockers, angiotensin-converting enzyme (ACE) inhibitors or calcium antagonists; hypoglycaemia due to oral antidiabetics; bleeding due to oral anticoagulants and bradycardia due to digoxin [4]. The use of a trigger tool focusing on clinical events and drugs frequently associated with such events may, therefore, reduce the problem of undiagnosed ADRs.

Several trigger tools have been developed to increase ADR detection in patient care. The most commonly known trigger tool is the Global Trigger Tool [11, 12], but other trigger tools targeting ADR detection, especially in the older population, have been investigated [13–15]. These trigger tools have in common that they comprise lists of either clinical events (e.g. ‘hypotension’), the use of specific drugs or antidotes (e.g. ‘naloxone use’) or abnormal drug or laboratory values (e.g. ‘potassium < 2.9 mEq/L’, ‘digoxin level > 2 ng/L’). However, the positive predictive values (PPVs) of such triggers were generally low, which impedes their implementation in clinical practice to improve ADR detection in older people [12–15]. Consequently, no ‘gold standard’ to improve ADR detection in older people has yet been established.

The performance of trigger tools in detecting clinically relevant ADRs in older people may be improved by combining clinical events with drug classes frequently associated with such events. The Dutch national geriatric guideline on *‘polypharmacy optimisation in hospitalised older people’* provides a consensus-based trigger tool listing combinations of certain clinical events and associated drugs that frequently result in ADR-related hospital admissions in older people [16]. The guideline strongly recommends screening each patient aged 70 years and older with polypharmacy (≥ 5 drugs) admitted to the emergency department for potential ADRs by using this ADR trigger tool. However, the recommendation has not been substantiated by evidence supporting the use of such a trigger tool in clinical practice. Hence, evaluation of the performance of the ADR trigger tool in the above-mentioned guideline is warranted.

This study aimed to investigate the performance of the ADR trigger tool recommended by the Dutch geriatric guideline and the recognition by usual care of ADRs detected with the tool in patients with polypharmacy acutely admitted to our geriatric ward.

## Methods

### Setting and study population

The study population consisted of patients aged 70 years and older with polypharmacy acutely admitted to the geriatric ward at a 1000 bed tertiary university hospital in the Netherlands (University Medical Centre Utrecht). Admissions of patients to the geriatric ward through the emergency department (ED) in the period between 01-01-2011 and 01-08-2017 were extracted with SAS enterprise guide v7.1 from a pseudonymised hospital database. Based on the consecutive order of randomly assigned numbers for each patient, admission letters were manually screened to include approximately 350 patients aged ≥ 70 years with polypharmacy. Polypharmacy was defined as the chronic use of at least five prescription drugs excluding dermatological preparations at admission [16]. For patients with multiple hospital admissions during the study period, the first admission that met the inclusion criteria was selected. A patient’s first admission was selected to minimise interference of consecutive hospital admissions with the study outcomes. Patients with an incomplete record (i.e. no admission or discharge letter available) were excluded.

### Study procedures

Electronic health records (EHRs) from the ED on the day of admission were screened for trigger–drug combinations listed in the ADR trigger tool of the Dutch national geriatric guideline ‘*polypharmacy optimisation in hospitalised older people*’ (first publication 2017, last revision 2020) [16]. This consensus-based trigger tool was developed in accordance with literature listing ten clinical events (i.e. triggers) and their associated drug classes frequently resulting in ADR-related admissions in older people [16–18]. Next, a causality assessment was performed for all detected trigger–drug combinations. The admission and discharge letters were also screened for ADR recognition by the attending physicians.

#### Screening for trigger–drug combinations

For this study, the original ADR trigger tool from the Dutch guideline was explicated to reduce undesirable variations in interpretation when applied to EHRs. Modifications to the original ADR trigger tool were implemented at three levels prior to screening for trigger–drug combinations:

1. Triggers were specified if they represented clinical events which could be linked to different drug classes (e.g. specification of ‘disturbed serum glucose levels’ into ‘hypoglycaemia’ and ‘hyperglycaemia’).
2. Drug classes were further specified following the ATC classification system (e.g. specification of ‘diuretics’ into ‘thiazide diuretics’, ‘loop diuretics’ and ‘potassium sparing diuretics’).
3. Triggers were merged for clinical events that are difficult to distinguish and are used interchangeably in clinical practice. For instance, ‘fall’ was merged with the triggers ‘collapse/(orthostatic) hypotension/dizziness/syncope’. Especially in older patients, it is difficult to distinguish falls and syncope, because falls can be preceded by temporarily loss of consciousness due to cerebral hypoperfusion [19].

Modifications to the original ADR trigger tool were performed by two researchers with clinical experience in medical practice (WL, NN) and reviewed by a senior geriatrician/clinical pharmacologist (WK) with the intention to follow the original ADR trigger tool as closely as possible. Table 1 illustrates the original ADR trigger tool as published in the Dutch national geriatric guideline and the explicated ADR trigger tool used for this research.

**Table 1:** The original ADR trigger tool as published in the Dutch national geriatric guideline ‘*polypharmacy optimisation in hospitalised older people’* and the explicated ADR trigger tool used for this research

Original ADR trigger tool		Explicated ADR trigger tool	
Trigger	Associated drug	Trigger	Associated drug
1. Fracture/fall^a^	A. Steroids B. Psychotropic agents^a^ C. Antihypertensive agents^a^	1. Fracture	A. Systemic corticosteroids
2. Collapse/hypotension/dizziness	A. Cardiac therapy (antihypertensive and antiarrhythmic agents) B. Psychotropic agents	2. Fall^a^/collapse/(orthostatic) hypotension/dizziness/syncope	A. Antihypertensive agents^a^: ACE-I, ARB, calcium antagonists, beta-blockers, thiazide diuretics, loop diuretics, potassium sparing diuretics, alpha-1-blockers, long-acting nitrates Antiarrhythmic agents: digoxin, class I, II and III antiarrhythmics B. Psychotropic drugs^a^: benzodiazepines, antipsychotics, antidepressants (i.e. SSRI, TCA and miscellaneous: duloxetine, venlafaxine and mirtazapine)
3. Bleeding (mostly gastrointestinal)/INR above therapeutic range	A. Anticoagulants B. Thrombocyte aggregation inhibitors C. NSAIDs	3.1 Gastrointestinal bleeding 3.2 Intracranial bleeding 3.3 Other bleedings	A. Vitamin K antagonists, DOACs, heparins, other anticoagulants B. Thrombocyte aggregation inhibitors C. NSAIDs
		3.4 Supratherapeutic INR	A. Vitamin K antagonists
4. Electrolyte disturbances/dehydration^b^	A. Diuretics B. ACE-I, ARB C. NSAIDs^b^ D. Antidepressants	4.1 Hyponatraemia	A. Thiazide diuretics, loop diuretics, potassium sparing diuretics B. ACE-I, ARB C. Antidepressants (i.e. SSRI, TCA and miscellaneous: duloxetine, venlafaxine and mirtazapine)
		4.2. Hypokalaemia	A. Thiazide diuretics, loop diuretics
		4.3. Hyperkalaemia	A. Potassium sparing diuretics B. ACE-I, ARB
5. Renal insufficiency	A. ACE-I, ARB B. NSAIDs	5. Renal insufficiency and/or dehydration^b^	A. ACE-I, ARB B. NSAIDs^b^ C. Thiazide diuretics, loop diuretics, potassium sparing diuretics
6. Disturbed serum glucose levels	A. Blood glucose lowering agents B. Corticosteroids	6.1 Hypoglycaemia	A. Oral antidiabetics, insulin and analogues
		6.2 Hyperglycaemia	B. Systemic corticosteroids
7. Heart failure	A. NSAIDs	7. Acute heart failure	A. NSAIDs
8. Constipation/ileus	A. Opioids B. Calcium channel blockers	8. Constipation/ileus (based on constipation)	A. Opioids B. Calcium channel blockers
9. Vomiting/diarrhoea	A. Antibiotics	9. Vomiting/diarrhoea	A. Antibiotics
10. Delirium/confusion/drowsiness	A. Cardiac therapy B. Psychotropic agents C. Benzodiazepines D. Urinary antispasmodic agents	10. Delirium/confusion/drowsiness	Drugs with anticholinergic and sedative properties (Supplementary Information 1), digoxin, anti-Parkinson drugs

*ACE-I* angiotensin-converting enzyme inhibitors, *ARB* angiotensin II receptor blockers, *SSRI* selective serotonin reuptake inhibitor, *TCA* tricyclic antidepressant, *DOAC* direct oral anticoagulant, *NSAIDs* non-steroidal anti-inflammatory drugs

^a^The trigger ‘fall’ and its associated drug classes ‘psychotropic agents’ and ‘antihypertensive agents’ were merged in the explicated version of the ADR trigger tool with the trigger ‘collapse…’

^b^The trigger dehydration and its associated drug class ‘NSAIDs’ was merged in the explicated version of the ADR trigger tool with the trigger ‘renal insufficiency’

Two researchers (WL, NN) screened EHRs for the presence of trigger–drug combination. The trigger had to be either documented as a symptom, or listed by the physician as a diagnosis or health problem. Trigger–drug combinations were regarded as discrete events if the prescribed drugs were related to different drug classes according to the explicated trigger tool. However, if multiple drugs from the same drug class were linked to the same trigger, this was counted as one trigger–drug combination. For example, oxycodone and morphine linked to constipation were considered as one trigger–drug combination (constipation-opioids), while hydrochlorothiazide (thiazide diuretics) and furosemide (loop diuretics) linked to hyponatraemia were considered as two separate trigger–drug combinations.

#### Causality assessment

A causality assessment was performed to establish the likelihood of an ADR for all trigger–drug combinations detected with the ADR trigger tool. Data from the admission and discharge letters were taken into account, because both letters could contain relevant information for causality assessment (e.g. to establish a potential time-relationship). A geriatrician (NN) and a clinical pharmacist (BS) independently assessed all trigger–drug combinations. The WHO-UMC system was used for causality assessment, which differentiates between the categories certain, probable, possible, unlikely and unclassifiable [20, 21]. Trigger–drug combinations with a causality score of certain, probable and possible were considered ADRs. Before the causality assessment, both appraisers trained with a previously published, Delphi-based chart review method developed to detect drug-related admissions by Thevalin et al.[22]. The level of agreement between the two appraisers was measured with the Cohen’s kappa test statistic (poor: κ < 0.00; slight: κ = 0.00–0.20; fair: κ = 0.21–0.40; moderate: κ = 0.41–0.60; substantial: κ = 0.61–0.80; almost perfect: κ = 0.81–1.00) [23]. If ratings differed ≥ 1 WHO-UMC category for causality between the two appraisers, the appraisers discussed each case to reach consensus. The appraisers consulted a third expert (WK, senior geriatrician-clinical pharmacologist) for a final consensus round in case no consensus was reached.

#### ADR recognition by usual care

In addition to the causality assessment, EHRs were screened for recognition of ADRs by usual care. Recognition was defined as an explicit documented trigger–drug combination by the attending physician (i.e. a geriatric resident, supervised by a geriatrician) in the admission and/or discharge letter, implying that the trigger–drug combination was identified as an ADR. In addition, explicit documentation of the trigger combined with medication changes in associated drugs (i.e. withdrawal, discontinuation or a dose adjustment) was also considered as being recognised by usual care.

### Outcomes

The performance of the ADR trigger tool was operationalised by calculating the overall PPV for detecting ADRs in general and for each trigger separately. The PPV was defined as the total number of detected trigger–drug combinations divided by the number of ADRs with a causality score of possible, probable or certain. The recognition by usual care was calculated for both ADRs with a causal relationship considered to be possible, probable or certain and for those with a probable or certain causal relationship.

### Data analysis

Descriptive data analysis and Cohen’s kappa test statistic was performed with IBM SPSS Statistics v.26.0.0.1.

## Results

### Study population

A random selection of 589 out of all 1366 patient admissions to the geriatric department through the ED between 01-01-2011 and 01-08-2017 was screened for eligibility. From this selection, 378 admissions met our inclusion criteria (i.e. age ≥ 70 and polypharmacy), of which 33 admissions were excluded because they were not a patient’s first admission within the study period. The study population of 345 patients had a median age of 84 (IQR 79–88). The median number of drugs at admission was 10 (IQR 8–13), and 61% of the patients were female. Subsequently, admission letters of these patients were screened for the presence of trigger–drug combinations according to the ADR trigger tool. Out of 345 eligible patients, 253 (73%) had at least one trigger–drug combination present. In 52% (178/345) of the total study population, at least one ADR with a causal relationship considered possible, probable or certain was present.

### Number of trigger–drug combinations

The total number of trigger–drug combinations was 941, with a median of 3 (IQR 2–5) and a maximum of 16 trigger–drug combinations per patient. Fall (32.4%), delirium (24.0%), renal insufficiency/dehydration (16.2%) and hyponatraemia (13.5%) were the most frequent clinical events and covered 86.3% of all identified trigger–drug combinations (Table 2).

**Table 2:** Number of trigger–drug combinations per trigger, mean number of associated drugs and results of the causality assessment

Trigger	Number of trigger–drug combinations, *n* (%)	Mean number of associated drugs per trigger (min–max)	Causality score, % (*n*)					PPV^a^, % (ADR/trigger–drug combinations)
			Unclassifiable	Unlikely	Possible	Probable	Certain	
Fall/collapse/(orthostatic) hypotension/dizziness/syncope	305 (32.4)	3.1 (1–8)	0 (0)	71.8 (219)	23.6 (72)	4.6 (14)	0 (0)	28.2 (86/305)
Delirium/confusion/drowsiness	226 (24.0)	2.3 (1–6)	3.1 (7)	73.9 (167)	16.4 (37)	5.3 (12)	1.3 (3)	23.0 (52/226)
Renal insufficiency and/or dehydration	152 (16.2)	1.8 (1–4)	0.7 (1)	37.5 (57)	43.4 (66)	17.8 (27)	0.7 (1)	61.8 (94/152)
Hyponatraemia	127 (13.5)	1.8 (1–4)	2.4 (3)	52.0 (66)	26.8 (34)	16.5 (21)	2.4 (3)	45.7 (58/127)
Constipation/ileus	35 (3.7)	1.3 (1–2)	2.9 (1)	28.6 (10)	31.4 (11)	20.0 (7)	17.1 (6)	68.6 (24/35)
Other bleedings	23 (2.4)	1.3 (1–2)	0 (0)	17.4 (4)	52.2 (12)	17.4 (4)	13.0 (3)	82.6 (19/23)
Hypoglycaemia	17 (1.8)	1.7 (1–2)	0 (0)	23.5 (4)	17.6 (3)	47.1 (8)	11.8 (2)	76.5 (13/17)
Hypokalaemia	14 (1.5)	1.0 (1–1)	0 (0)	0 (0)	64.3 (9)	28.6 (4)	7.1 (1)	100 (14/14)
Hyperkalaemia	13 (1.4)	1.3 (1–1)	0 (0)	30.8 (4)	46.2 (6)	23.1 (3)	0 (0)	69.2 (9/13)
Supratherapeutic INR	9 (1.0)	1.0 (1–1)	0 (0)	0 (0)	0 (0)	88.9 (8)	11.1 (1)	100 (9/9)
Vomiting/diarrhoea	9 (1.0)	1.0 (1–1)	0 (0)	11.1 (1)	22.2 (2)	44.4 (4)	22.2 (2)	88.9 (8/9)
Gastrointestinal bleeding	7 (0.7)	1.0 (1–1)	0 (0)	14.3 (1)	28.6 (2)	42.9 (3)	14.3 (1)	85.7 (6/7)
Hyperglycaemia	2 (0.2)	1.0 (1–2)	0 (0)	50.0 (1)	0 (0)	50.0 (1)	0 (0)	50.0 (1/2)
Fracture	1 (0.1)	1.0 (1–1)	0 (0)	100 (1)	0 (0)	0 (0)	0 (0)	0 (0/1)
Acute heart failure	1 (0.1)	1.0 (1–1)	0 (0)	100 (1)	0 (0)	0 (0)	0 (0)	0 (0/1)
Intracranial bleeding	0 (0.0)	N/A	0 (0)	0 (0)	0 (0)	0 (0)	0 (0)	N/A
Total	941 (100)	2.0 (1–8)	1.3 (12)	57.0 (536)	27.0 (254)	12.3 (116)	2.4 (23)	41.8 (393/941)

^a^The PPV was defined as the number of ADRs divided by the number of all detected trigger–drug combinations per trigger

## Causality assessment and PPV

Of the 941 identified trigger–drug combinations, 41.8% (*n* = 393) were adjudicated as an ADR by the two appraisers in 178 patients. More than a quarter (27.0%) of all 941 trigger–drug combinations were considered as possible ADRs, 12.3% were adjudicated as probable ADRs, and 2.4% as certain ADRs. In 57.0% of the trigger–drug combinations, an ADR was considered as unlikely, and the other 1.3% of the combinations were unclassifiable (Table 2). Inter-rater agreement for causality assessment of ADRs was substantial (κ = 0.61–0.80) with a Cohen’s kappa of 0.76 [23]. In total, causality scores of 163/941 trigger–drug combinations differed between the adjudicators, with a difference of only one WHO-UMC category in 91.1% of the cases (*n* = 149). The two appraisers reassessed and discussed all discrepancies and reached a consensus without consulting a third expert.

Overall, the PPV of the ADR trigger tool was 41.8%. The PPV varied considerably across triggers. The PPV related to the triggers fall (28.2%) and delirium (23.0%) were the lowest, whereas the mean number of drugs associated with these triggers was highest with a large range (fall: mean 3.1, min–max 1–8; delirium: mean 2.3, min–max 1–6). Although numbers were relatively small, the PPVs related to the triggers hypokalaemia (100%), supratherapeutic INR (100%) and vomiting/diarrhoea (88.9%) were highest (Table 2).

### Drugs related to ADRs

More than half of the 941 trigger–drug combinations detected by the ADR trigger tool were associated with three drug classes: diuretics (25.4%), agents acting on the renin–angiotensin system (16.7%) and psychotropic agents (12.2%). The top three drug classes most frequently associated with the 393 ADRs were diuretics (35.4%), agents acting on the renin–angiotensin system (13.5%) and analgesics (11.2%), covering 60% of all drugs that caused an ADR.

### ADR recognition by usual care

Usual care recognised 51.8% (481/929) of the trigger–drug combinations detected by the trigger tool and for which a causality classification could be determined. 42.3% (393/929) were considered ADRs with at least possible causality, of which 83.5% (328/393) were recognised by usual care according to information in the admission and discharge letters (Table 3). 16.5% (65/393) of ADRs were not recognised by usual care, of which 93.9% (*n* = 61) had a causal relationship considered to be possible. The majority of these possible ADRs not recognised by usual care were related to the top three most common events (fall, *n* = 29; delirium, *n* = 13; renal insufficiency, *n* = 10). Three probable ADRs were not recognised (furosemide–hyponatraemia; fentanyl–constipation; fentanyl–delirium) and one certain ADR was not recognised by usual care (bumetanide–renal insufficiency/dehydration). Recognition by usual care increased to 97.1% (135/139) when only ADRs considered to be probable or certain ADRs were included (Table 3).

**Table 3:** Number of ADRs per trigger and their associated drug classes, stratified for ADRs with a causal relationship considered to be possible, probable or certain, and for those considered to be probable or certain

Trigger	ADR causality score: possible–probable–certain			ADR causality score: probable–certain		
	ADR, *n* (%)	Drugs related to ADR (*n)*	Recognition by usual care, %	ADR, *n* (%)	Drugs related to ADR (*n*)	Recognition by usual care, %
Fall/collapse/(orthostatic) hypotension/dizziness/syncope	86 (21.9)	Diuretics (17)	66.3	14 (10.1)	Diuretics (4)	100
		Beta blocking agents (17)			Psychoanaleptics (3)	
		Agents acting on RAAS (17)			Beta blocking agents (2)	
		Other (35)			Other (5)	
Delirium/confusion/drowsiness	52 (13.2)	Analgesics (21)	75.0	15 (10.8)	Analgesics (12)	93.3
		Psycholeptics (9)			Antiepileptics (1)	
		Psychoanaleptics (7)			Anti-parkinson drugs (1)	
		Other (15)			Cardiac therapy (1)	
Renal insufficiency and/or dehydration	94 (23.9)	Diuretics (59)	89.4	28 (20.1)	Diuretics (12)	96.4
		Agents acting on RAAS (27)			Agents acting on RAAS (9)	
		Antiinflammatory and –rheumatic drugs (8)			Antiinflammatory and -rheumatic drugs (7)	
Hyponatraemia	58 (14.8)	Diuretics (44)	96.6	24 (17.3)	Diuretics (18)	95.8
					Psychoanaleptics (5)	
		Psychoanaleptics (9)			Agents acting on RAAS (1)	
		Agents acting on RAAS (5)				
Constipation/ileus	24 (6.1)	Analgesics (23)	91.7	13 (9.4)	Analgesics (13)	92.3
		Calcium channel blockers (1)				
Other bleedings	19 (4.8)	Antithrombotic agents (17)	73.7	7 (5.0)	Antithrombotic agents (7)	100
		Antiinflammatory and antirheumatics (2)				
Hypoglycaemia	13 (3.3)	Drugs used in diabetes (13)	100	10 (7.2)	Drugs used in diabetes (10)	100
Hypokalaemia	14 (3.6)	Diuretics (14)	92.9	5 (3.6)	Diuretics (5)	100
Hyperkalaemia	9 (2.3)	Diuretics (5)	88.9	3 (2.2)	Diuretics (2)	100
					Agents acting on RAAS (1)	
		Agents acting on RAAS (4)				
Supratherapeutic INR	9 (2.3)	Antithrombotic agents (9)	100	9 (6.5)	Antithrombotic agents (9)	100
Vomiting/diarrhoea	8 (2.0)	Antibacterials for systemic use (8)	87.5	6 (4.3)	Antibacterials for systemic use (6)	100
Gastrointestinal bleeding	6 (1.5)	Antithrombotic agents (6)	83.3	4 (2.9)	Antithrombotic agents (4)	100
Hyperglycaemia	1 (0.3)	Corticosteroids for systemic use (1)	100	1 (0.7)	Corticosteroids for systemic use (1)	100
Fracture	0 (0)	N/A	N/A	0 (0)	N/A	N/A
Acute heart failure	0 (0)	N/A	N/A	0 (0)	N/A	N/A
Intracranial bleeding	0 (0)	N/A	N/A	0 (0)	N/A	N/A
Total	393 (100)	Diuretics (139)	83.5	139 (100)	Diuretics (41)	97.1
		Agents acting on RAAS (53)			Antithrombotic agents (20)	
		Analgesics (44)			Analgesics (25)	
		Other (157)			Other (53)	

*RAAS* renin–angiotensin–aldosterone

In 75.6% of possible, probable or certain ADRs and in 85.6% of probable or certain ADRs, the suspected drug was discontinued, or the dosage was reduced by usual care. The top three most frequently discontinued drugs related to ADRs were thiazides, opioids and high-ceiling diuretics. Table 3 provides a detailed overview of the number of ADRs per trigger and their associated drug classes in relation to their recognition by usual care. ADRs were stratified for a causal relationship considered to be possible, probable or certain and for those considered to be probable or certain.

## Discussion

### Main findings

ADRs were highly prevalent in older patients with polypharmacy acutely admitted to the geriatric ward. The ADR trigger tool detected one or more trigger–drug combinations at admission in almost three quarters (73%) of all screened patients, and more than half (52%) of these patients had at least one confirmed ADR after causality assessment. The overall PPV of the ADR trigger tool was 41.8%, indicating that less than half of the trigger–drug combinations were considered to be ADRs. Usual care recognised the majority of ADRs (83.5%), increasing to 97.1% when restricted to possible and certain ADRs.

### Performance

The performance of the ADR trigger tool recommended by the Dutch geriatric guideline was not previously studied. Using an ADR trigger tool may be a helpful and efficient strategy to increase ADR detection in older people, especially in cases of low recognition by usual care. A high PPV is important for a positive balance between reviewing signals and detecting actual ADRs. Although there is no generally accepted definition to distinguish ‘good’ from ‘poor’ trigger tool performance—which also depends on its intended use—a PPV ≥ 20% is often considered good [23, 24]. In our study, the PPV per trigger of the investigated ADR trigger tool was highly variable, ranging from 0–100%. However, if triggers with a frequency of only one were excluded, all triggers had a PPV ≥ 20%, of which the PPVs for the triggers ‘fall/…/dizziness’ (PPV 28%) and ‘delirium/…/drowsiness’ (PPV 23%) were lowest. These clinical events often have multiple possible causes related to comorbidity, drugs and drug combinations, impeding the confirmation of a clear causal relationship. The mean number of drugs related to these two events at a patient’s level were highest. In contrast, trigger–drug combinations based on clinical events related to a single drug class (e.g. vitamin K antagonist–supratherapeutic INR) or for which a dechallenge usually results in a direct improvement (e.g. diuretics–hypokalaemia) were more likely considered to be ADRs.

The low PPV for triggers related to fall and delirium are in line with other findings. Carnevali et al. found a PPV for the triggers ‘fall’ and ‘emergence of confused state’ of 19% and 9%, respectively, in hospitalised adults [12]. In addition, a French retrospective cohort study in acutely admitted geriatric patients investigated the triggers ‘fall’ and ‘delirium’ from the Global Trigger Tool [11, 25]. The mean number of suspected drugs per patient related to these clinical events was comparable with our results, as well as the PPV for delirium (21% vs 23%). However, the PPV for falls was much higher (54% vs. 28%), which is likely due to differences in the ADR causality method used; the relationship between the suspected drug and the identified ADRs in this French study was uncertain in over 80%. Removing the triggers for falls and delirium from the ADR trigger tool will increase the overall PPV of the ADR trigger tool from 41.8% to 62.2% (*n* = 255/410, Table 2). Nevertheless, we would not recommend excluding falls and delirium as triggers because these clinical events are often associated with drug-related admissions in older patients with polypharmacy [24]. In addition, a large proportion of ADRs would be excluded (35%, *n* = 138/393), and recognition by usual care for these triggers was lowest for ADRs of at least possible causality (Table 3). To increase the PPV, we would rather suggest to explore strategies for excluding drugs with a relatively low risk on the clinical event. A recent observational study compared the association of potentially inappropriate medication on inpatient falls listed in the explicit screening tools STOPP v2, STOPP v2 section K, and STOPPFall [26–28] Although all screening tools were independently associated with falls, the strongest effect was identified for STOPP section K [28]. This is plausible because STOPP section K is the most restrictive tool, including only four drug classes with highest risk of falls (i.e. benzodiazepines, hypnotic z-drugs, vasodilator drugs, and neuroleptic drugs). For delirium, selecting drugs with the highest anticholinergic burden will likely increase the PPV. However, a disadvantage of excluding drugs from the ADR trigger tool is that less ADRs may be detected.

The difficulties in achieving a high PPV in ADR detection were illustrated in a systematic review on methods to detect drug-related problems. This systematic review identified 28 studies, three of which used a trigger tool to detect ADRs [29]. The PPVs of these ADR trigger tools ranged from 1.8% to 32% [30–32]. The study with the lowest PPV (1.8%) was the only one performed in a geriatric population (rehabilitation ward) using a commercially available database grounded on potential ADRs extracted from a drug’s product information [30]. The highest PPV was reported in patients (age 16–90 years) admitted to a gastroenterology department using a trigger tool solely based on laboratory signals [32]. The use of trigger tools appeared to be the most labor-efficient method; however, incident report review generally showed a higher specificity compared to other methods.

More recently, Zerah et al. evaluated the PPV of a trigger tool to detect adverse drug events (ADEs) and drug-related admissions (DRAs) in older people based on chart review [24]. The DRA trigger tool comprised 26 triggers and associated drugs frequently involved in ADEs. The DRA trigger tool was more comprehensive than the ADR trigger tool used in our study and included triggers to detect ADEs, including both ADRs and medication errors (i.e. underuse, overuse and misuse of drugs). The overall PPV for the detection of ADEs of the DRA trigger tool was 87% [24]. The better performance of the DRA trigger tool compared with the ADR trigger tool may be explained by the inclusion of medication errors, which had a large impact on the PPV. For instance, 11.8% (*n* = 76) of all ADEs with a causal relationship were related to the trigger ‘heart failure’, with the majority of these ADEs being adjudicated as underuse of beta-blockers, ACE-inhibitors, and diuretics [24]. For this reason, the PPVs of these two tools are difficult to compare.

### ADR recognition by usual care

In addition to aiming for a high PPV, an ADR trigger tool needs to be of clinical value to usual care and increase the detection of unrecognised ADRs. Previous studies reported that drug-related problems are missed or misdiagnosed in approximately 40–60% of the cases by physicians at the ED; however, we found a much higher recognition by usual care of ADRs identified with the use of the ADR trigger tool [6–8]. There are several explanations for this discrepancy. First, we investigated a subset of most frequent and serious ADRs in older people targeted by the ADR trigger tool, which cannot be compared with the broader definition of ‘drug-related problems’ in previous studies. In addition, our study was performed in an academic, teaching hospital and all patients were under geriatric care. Compared to other specialists, geriatric residents are well trained in detecting drug-related problems in their patients under the direct supervision of experienced geriatricians [33]. The high recognition of ADRs found in our study was comparable with the results of Klopotowska et al. who found that 80% of ADRs of at least possible causality in older hospitalised patients admitted to an internal medicine ward were recognised by usual care during the hospital stay [34]. Similar to our results, the majority of unrecognised ADRs were those with a possible causality score [34].

### Strengths and limitations

If implemented in daily practice, the PPV as a measure for performance is an important outcome to assess the relevance of triggers. The reported ADR recognition by usual care is highly relevant in deciding whether implementation of such a tool would add clinical value to usual patient care.

To ensure that ADR recognition by usual care was not biased, we selected patients who were admitted before publication of the tool in the national guideline. Two independent clinicians thoroughly and manually screened admission letters for trigger–drug combinations, followed by causality assessment by a geriatrician and a clinical pharmacist revealing substantial inter-rater agreement (κ = 0.76).

There are, however, several limitations to this research. First, EHRs were only screened for trigger–drug combinations listed in the ADR trigger tool. Therefore, the negative predictive value, sensitivity or specificity of the tool could not be calculated. Second, retrospective studies based on chart review rely on documented information by attending physicians. The introduction of information bias by physician’s notes and actions cannot be fully ruled out. For instance, the screening of trigger–drug combinations was based on information documented in admission letters and laboratory results were not examined as a primary source of triggers. A mild hyponatraemia with concomitant use of diuretics could potentially have been missed as trigger–drug combination if it was not mentioned as a clinical problem by the attending physician. However, the triggers listed in the ADR trigger tool are serious and admission letters were comprehensive, which makes underreporting of these triggers unlikely.

Third, the definition of ‘recognition by usual care’ was not very specific since a documented event combined with discontinuation or a dose adjustment of the associated drug was also considered as being ‘recognised’ without explicit mention. However, this does not necessarily correspond with ADR recognition because drugs could be discontinued for other reasons (e.g. a lack of indication). In addition, the persistence of drug changes after hospital discharge was not evaluated in our study. A discontinuation or dose adjustment of the suspected drug was implemented by the attending physician in three quarters of ADRs, but previous research illustrated that a quarter of drugs discontinued because of an ADR were re-prescribed after admission [35].

In addition, this study was performed in a specific population of older patients with polypharmacy acutely admitted to a geriatric ward. The admission to a geriatric ward in an academic, teaching hospital could have biased the type and prevalence of certain trigger–drug combinations. For instance, patients presenting with fall and delirium are likely to be admitted to a geriatric ward; these clinical events were most prevalent in our population comprising more than half of all identified trigger–drug combinations. Consequently, these two triggers had the largest impact on the overall PPV of the ADR trigger tool. In contrast, the clinical event ‘intracranial bleeding’ was absent in our population and, thus, had no impact on the overall PPV. Acutely admitted patients with an intracranial bleeding are more likely to be admitted to a neurosurgical ward instead of a geriatric ward. Furthermore, geriatric residents and their supervisors in an academic, teaching hospital may be more focused on ADR recognition compared to other medical specialties. For these reasons, the generalisation of ADR prevalence and ADR recognition are limited. Lastly, the PPV was not stratified for different patient populations because the availability of baseline patient characteristics was limited.

### Implications

The ADR trigger tool detected ADRs in more than half (52%) of all patients with polypharmacy acutely admitted to the geriatric ward. Combining the ADR trigger tool with ADR risk-prediction models may be a good future strategy to identify older patients at highest risk of ADRs, potentially increasing the predictive value of the tool. However, currently available ADR risk-prediction models for use in older people, such as the GerontoNet ADR risk scale and the Adverse Drug Reaction Risk in Older Persons (ADRROP) prediction scale, failed to predict ADRs well, and the most important risk factor for the occurrence of ADRs—polypharmacy—was already included in our study [10, 36–38].

ADR recognition by geriatric residents/geriatricians was very high for ADRs detected with the trigger tool in the setting of a tertiary university teaching hospital. Therefore, implementation of this trigger tool is not likely to improve care for older patients acutely admitted to our geriatric ward. However, ADR recognition by physicians less experienced in ADR detection in older people may be lower. Future research could focus on the clinical value of the tool if used in older patients acutely admitted to non-geriatric wards. In addition, it would be interesting to investigate if the ADR trigger tool could decrease the time to ADR detection, for example, when integrated with electronic healthcare systems. The use of clinical decision support systems to improve in-hospital fall and delirium care (e.g. reminders for patient screening and support to review medication) was identified as a facilitator in a recent interview study among Dutch healthcare professionals [39]. However, the risk of alert fatigue was also addressed as a potential barrier for this strategy [39]. In view of our results, we highly recommend conducting performance and feasibility studies before recommending ADR trigger tools as a standard of care.

## Conclusion

The ADR trigger tool has predictive value (PPV 41.8%), but implementation of this tool is not likely to improve ADR recognition in older patients acutely admitted to our geriatric ward because the majority of ADRs were recognised by usual care.

## Supplementary Information

Below is the link to the electronic supplementary material.

Supplementary file1 (PDF 289 KB)
