# Supplementary material for: Performance of a trigger tool for detecting adverse drug reactions in patients with polypharmacy acutely admitted to the geriatric ward
Source: Eur Geriatr Med. 2022 May 30;13(4):837–47. doi: 10.1007/s41999-022-00649-x (PMC9378479; doi:10.1007/s41999-022-00649-x)
Supplement: Supplementary file 1 — Supplementary file1 (PDF 289 KB) [file 41999_2022_649_MOESM1_ESM.pdf]

Supplementary Information SI1

Specification of drugs with anticholinergic and sedative properties, digoxin and anti-Parkinson drugs associated with the trigger for delirium/confusion/drowsiness. The list of drugs with anticholinergic and sedative properties available in the Netherlands was based on publications of Hilmer et al.[1] and Duran et al.[2]

**Drugs**	**ATC code**	**Drugs**	**ATC code**
**Benzodiazepine agonists**		**Antidepressants**	
Diazepam	N05BA01	Venlafaxine	N06AX16
Oxazepam	N05BA04	Mirtazapine	N06AX11
Clorazepate	N05BA05	Paroxetine	N06AB05
Temazepam	N05CD07	Sertraline	N06AB06
Alprazolam	N05BA12	Citalopram	N06AB04
Lorazepam	N05BA06	Escitalopram	N06AB10
Zolpidem	N05CF02	Phenelzine	N06AF03
Zopiclone	N05CF01	Amitryptiline	N06AA09
Bromazepam	N05BA08	Clomipramine	N06AA04
Flurazepam	N05CD01	Nortriptyline	N06AA10
Prazepam	N05BA11	Fluoxetine	N06AB03
Nitrazepam	N05CD02	Trazodone	N06AX05
Lormetazepam	N05CD06		
Brotizolam	N05CD09	**Antipsychotics**	
		Risperidone	N05AX08
**Opiod analgesics**		Quetiapine	N05AH04
Fentanyl	N02AB03	Olanzapine	N05AH03
Morphine	N02AA01	Haloperidol	N05AD01
Tramadol	N02AX02	Clozapine	N05AH02
Oxycodone	N02AA05	Pipamperon	N05AD05
Codeine	N02AA59	Zuclopenthixol	N05AF05
Buprenorphine	N02AE01		
		**Urinary antispasmodics**	
**Anti-epileptics**		Oxybutynin	G04BD04
Phenytoin	N03AB02	Tolterodine	G04BD05
Carbamazepine	N03AF01	Darifenacine	G04BD10
Oxcarbazepine	N03AF02	Solifenacin	G04BD08
Valproic acid	N03AG01	Fesoterodine	G04BD11
Gabapentin	N03AX12		
Lamotrigine	N03AX09	**Anticholinergic bronchodilators**	
Levetiracetam	N03AX14	Ipratropium	R03BB01
Clonazepam	N03AE01	Tiotropium	R03BB04
Pregabalin	N03AX16		
		**Miscellaneous drugs**	
**Antihistamines**		Tamsulosin	G04CA02
Levocetirizine	R06AE09	Doxazosin	C02CA04
Fexofenadine	R06AX26	Disopyramide	C01BA03
Cinnarizine	N07CA02	Loperamide	A07DA03
Hydroxyzine	N05BB01	Levomepromazine	N05AA02
Cetirizine	R06AE07	Clonidine	C02AC01
Clemastine	R06AA04	Methyldopa	C02AB01
**Digoxin**	C01AA05	**Anti-Parkinson drugs**	N04

[1] Hilmer, S. N. *et al.* A Drug Burden Index to Define the Functional Burden of Medications in Older People. *Am. Geriatr. Soc.* **167,** 781–787 (2007). [2] Durán, C. E., Azermai, M. & Stichele, R. H. Vander. Systematic review of anticholinergic risk scales in older adults. *Eur. J. Clin. Pharmacol.* **69,** 1485–1496 (2013).

ATC = anatomical therapeutic chemical classification

This supplementary information relates to:
Nikki MF Noorda et al. UMC Utrecht, the Netherlands. Performance of a Trigger Tool for Detecting Adverse Drug Reactions in Patients with Polypharmacy Acutely Admitted to the Geriatric Ward. European Geriatric Medicine. Email: nikkinoorda@hotmail.com
